# Improved Microbiological Diagnosis of Bone and Joint Infections Using Mechanical Bead-Milling Extraction of Bone Specimens with the Ultra-Turrax^®^ System

**DOI:** 10.3390/diagnostics16020309

**Published:** 2026-01-18

**Authors:** Maxime Brunaud, Adeline Boutet-Dubois, Alix Pantel, Florian Salipante, Rémy Coulomb, Albert Sotto, Jean-Philippe Lavigne, Nicolas Cellier

**Affiliations:** 1 Service d’Orthopédie, University of Montpellier, CHU Nîmes, 30900 Nîmes, France; brunaudmaxime@gmail.com (M.B.); remy.coulomb@chu-nimes.fr (R.C.); nicolas.cellier@chu-nimes.fr (N.C.); 2 Service de Microbiologie et Hygiène Hospitalière, VBIC, INSERM U1047, University of Montpellier, CHU Nîmes, 30900 Nîmes, France; adeline.dubois@chu-nimes.fr (A.B.-D.); alix.pantel@chu-nimes.fr (A.P.); 3 Service BESPIM, University of Montpellier, CHU Nîmes, 30900 Nîmes, France; florian.salipante@chu-nimes.fr; 4 Serive de Maladies Infectieuses et Tropicales, VBIC, INSERM U1047, University of Montpellier, CHU Nîmes, 30900 Nîmes, France; albert.sotto@chu-nimes.fr

**Keywords:** bone and joint infections, prosthetic joint infection, Ultra-Turrax^®^, bead-milling homogenization, microbiological diagnosis, pre-analytical processing

## Abstract

**Background**: Accurate microbiological diagnosis of bone and joint infections (BJIs) is frequently hampered by low bacterial load, biofilm formation, and suboptimal tissue processing. This study evaluated the diagnostic performance of mechanical bead-milling using the Ultra-Turrax^®^ Tube Drive system compared with standard vortex homogenization. **Methods**: In a prospective cohort of 116 patients undergoing surgery for suspected BJIs, 540 intraoperative samples were processed using both methods. Culture and 16S rRNA PCR results were analyzed using classical and Bayesian statistical approaches. Diagnostic performance was assessed globally and across specimen types and anatomical sites. **Results**: Ultra-Turrax^®^ significantly improved sensitivity across all sample types (87.1% vs. 75.2%, *p* < 0.0001), while maintaining comparable specificity (>99%). Culture positivity increased by 17%, with the greatest gains observed in bone samples and hip prosthesis infections. Quantitative cultures demonstrated a 1.5–2 log_10_ CFU/mL increase in bacterial yield. In culture-negative specimens, 16S rRNA PCR detection doubled with Ultra-Turrax^®^ processing (26% vs. 13%, *p* = 0.04). No increase in contamination was observed. Time to positivity was similar between methods, although Ultra-Turrax^®^ provided earlier results in 17% of cases. Bayesian modeling confirmed superior sensitivity (posterior probability > 0.995). **Conclusions**: Ultra-Turrax^®^ bead-milling markedly enhances microbiological detection in BJIs, particularly in low-biomass and bone-derived specimens. Its simplicity, reproducibility, and compatibility with routine workflows support its integration into diagnostic pathways. This pre-analytical optimization may improve etiological identification and guide more targeted antimicrobial therapy.

## 1. Introduction

Bone and joint infections (BJIs), particularly those involving prosthetic joints or osteosynthetic materials, are severe conditions associated with high morbidity, functional impairment, and substantial healthcare costs [1,2]. Their rising incidence, complex management, and potentially devastating outcomes make BJIs among the most challenging bacterial infections to treat [3,4]. These infections often lead to irreversible bone destruction, long-term disability, and reduced quality of life, contributing to a considerable social and economic burden [5,6].

The number of primary and revision arthroplasties, as well as fracture repairs involving implanted hardware, has steadily increased over the past two decades, a trend expected to continue [6]. In France alone, 28,365 infectious episodes were reported between 2014 and 2019, including 17,328 classified as complex BJIs [7].

Timely and accurate microbiological diagnosis is essential for effective management and targeted antimicrobial therapy. However, conventional culture-based methods are often slow and lack sensitivity, particularly in biofilm-associated infections or polymicrobial communities with fastidious organisms [8,9,10]. These limitations frequently result in culture-negative cases or misclassification as aseptic failures, even after prolonged incubation, leading to delayed or inappropriate treatment.

To improve bacterial recovery, several pre-analytical tissue processing techniques have been explored, including bead-milling and sonication of explanted devices [11,12,13]. Bead-milling mechanically disrupts tissues using high-speed agitation of small beads, effectively releasing bacteria embedded in dense matrices such as bone [14]. Among available systems, the Ultra-Turrax^®^ Tube Drive (IKA-Werke GmbH; Staufen, Germany) offers semi-automated, reproducible homogenization for bone biopsies. While preliminary studies suggest variable benefits for soft tissue, evidence supporting its use for bone samples remains limited [14,15].

The primary aim of this study was to evaluate whether semi-automated bead-milling using the Ultra-Turrax^®^ system improves microbiological diagnosis of BJIs compared to standard manual homogenization. We assessed diagnostic performance, microbial diversity, sample-specific yield, and pathogen recovery.

## 2. Materials and Methods

### 2.1. Study Design

This prospective observational study was conducted at Nîmes University Hospital (France) between May 2016 and November 2018. The protocol was approved by a national ethics committee (CCP Sud Méditerranée III—approval date: 21 September 2015; registration number: LOCAL/2015/NC-01-2015-A00720-49) and registered on ClinicalTrial.gov (NCT02598141). Written informed consent was obtained from all participants or their legal representatives.

### 2.2. Patient Inclusion

Adults (≥18 years) undergoing surgery for suspected BJIs in the presence of prosthetic or osteosynthetic material were eligible. Exclusion criteria included pregnancy, absence of implant material, aseptic revision, recent surgery (≤3 months) at the same site, antibiotic therapy within 15 days prior to surgery, or concurrent enrollment in another interventional trial.

### 2.3. Clinical and Biological Data

Baseline data included demographic characteristics (age, sex, weight, body mass index (BMI), prior antibiotic use, infection site, infection type (early, delayed, chronic, recurrent), presence and location of prothesis, type of osteosynthesis material, surgical procedures, and presence of fistula and/or purulence in contact with the prosthesis. Laboratory data included complete blood count, leukocyte and neutrophil counts, and C-reactive protein levels.

### 2.4. Sample Collection

During surgery, five bone or soft-tissue samples were collected from periprosthetic or infected areas. Specimens for culture were obtained via transcutaneous bone or tissue biopsy and/or fluid aspiration by trained orthopedic surgeons following standardized procedures [16]. Peripheral blood cultures were drawn if the patient’s temperature exceeded 38 °C.

Each specimen was divided into two aliquots: one placed in a sterile container for manual homogenization (Standard method) [16], and the other placed in an irradiated plastic vial (15 mL) containing 2 mL sterile water and 10 stainless-steel beads for bead-milling homogenization (Ultra-Turrax^®^ method). Synovial fluid samples were processed in parallel and inoculated into BD BACTEC^TM^ PLUS Aerobic and into BD BACTEC^TM^ Lytic Anaerobic bottles (Becton Dickinson, Le Pont-de-Claix, France) when available [16]. All samples were labeled and transported immediately to the microbiology laboratory under institutional protocols.

### 2.5. Microbiological Processing

For the standard method, samples were processed under a biosafety cabinet using aseptic techniques [16]. After adding 6–8 drops of Trypticase-soja broth (bioMérieux, Marcy l’Etoile, France), samples were manually homogenized with a single-use scalpel. For the Ultra-Turrax^®^ method, sealed vials were placed in the Ultra-Turrax^®^ Tube Drive system, which magnetically accelerated the beads for 5 min at 6000 rpm. Blood cultures were incubated in the BD BACTEC^TM^ FX Blood Culture System (Beckton Dickinson).

Both aliquots were inoculated onto aerobic blood agar, chocolate agar, and anaerobic blood agar plates, and into Schaedler broth (bioMérieux). Agar plates were incubated at 35 ± 2 °C for 7 days, while Schaedler broth was incubated for 14 days. Turbid broths were subcultured onto aerobic and anaerobic blood agar plates. Synovial fluids were similarly processed, with 0.1 mL inoculated onto agar plates and Schaedler broth.

Bacterial identification was performed by MALDI-TOF MS (Vitek^®^ MS, bioMérieux). Antibiotic susceptibility testing was conducted using the Vitek^®^ 2 system (bioMerieux) or disk diffusion on Muller-Hinton agar, following CA-SFM/EUCAST guidelines. Remaining material was stored at −80 °C for 16S rRNA PCR in culture-negative cases [17].

### 2.6. Reference Standard and Statistical Analysis

Infections were defined using the 2018 EBJIS (European Bone and Joint Infection Society) criteria [18]. Comparisons between methods were based on microbiological outcomes. Given the absence of a perfect gold standard, a Bayesian latent class model was used to jointly estimate the performance of both methods using prior information from literature and expert consensus. According to French Infectious Diseases Society guidelines [19], a patient was considered positive if skin microbiota bacteria were isolated from ≥ 3 of 5 samples. For non-skin microbiota bacteria, detection in a single sample was sufficient. Patients with indeterminate results were classified as non-infected. The reference classification was binary: “Positive” (confirmed infection) and “Negative” (infection excluded or not detectable, interpreted in clinical context).

Statistical analyses were performed using R (version 4.1.0) and OpenBUGS (version 3.2.3). Continuous variables were reported as means ± standard deviations (SD) or medians with interquartile ranges, and categorical variables as frequencies and percentages. All tests were two-sided with a significance threshold of 0.05.

For the primary endpoint, traditional diagnostic comparisons assume a perfect reference standard. In this study, the conventional reference method, standard bacteriological processing, is imperfect for detecting prosthetic joint infection. We hypothesized that Ultra-Turrax^®^ homogenization improves diagnostic sensitivity. To address the absence of a true gold standard, a Bayesian latent class model was applied to estimate sensitivities, specificities of both diagnostic methods (standard vs. Ultra-Turrax^®^), and disease prevalence (latent variable representing true infection status). A conditionally dependent fixed-effects model was used with priors: prevalence > 90%; sensitivity ≈ 60%, and specificity >98% for the standard method; expected sensitivity gain of ~20% (≈72%) for Ultra-Turrax^®^. Non-informative priors were used for sensitivities. Posterior distributions were estimated via Markov Chain Monte Carlo (MCMC) simulations using Gibbs sampling (OpenBUGS via the R package R2OpenBUGS (Version 3.2.3)). Convergence was assessed graphically and with Gelman–Rubin and Geweke diagnostics. Results were reported as medians with 95% credible intervals (CrI). Sensitivities and specificities were compared using posterior distribution tests and Bayesian *p*-values. Sensitivity was prioritized in case of discrepancies between methods.

A frequentist analysis was also performed using a composite reference standard combining microbiological, clinical, and laboratory findings (e.g., pus, fistula, elevated CRP or neutrophils), in accordance with SPILF recommendations [19].

Secondary endpoints included bacterial species prevalence and culture positivity by method, compared using Chi-square, McNemar, or Fisher’s exact tests. Subgroup analyses were conducted by sample type, joint type, and implant type, using the same methodology as for the primary endpoint.

## 3. Results

### 3.1. Study Population

A total of 116 patients were enrolled, yielding 540 intraoperative samples. Demographic and clinical characteristics are summarized in Table 1. The mean age was 67.9 ± 13.6 years, and 51% of patients were male (*n* = 59). Sample types included bone fragments (*n* = 192), soft tissue (*n* = 173), and implant-associated specimens (*n* = 175).

### 3.2. Overall Diagnostic Performance

Across all specimen types, Ultra-Turrax^®^ showed higher sensitivity than the standard method while maintaining comparable specificity (Table 2). In the frequentist analysis, sensitivity was 87.1% [95% CI: 80.6–93.7] for Ultra-Turrax^®^ versus 75.2% [66.8–83.7] for the standard method. Bayesian inference confirmed this improvement, with estimated sensitivities of 77.1% [95% CrI 68.4–86.1%] for Ultra-Turrax^®^ vs. 66.7% [57.4–76.3] for the standard technique, and a posterior probability > 0.99 in favor of Ultra-Turrax^®^. Both models confirmed statistical significance (*p* < 0.0001). Specificity remained high and equivalent between methods: 100% [95% CI: 100–100] for both in the frequentist analysis, and 99.2% [95% CrI: 95.7–99.9] for the standard method and 99.1% [95% CrI: 95.3–99.9] for Ultra-Turrax^®^ in the Bayesian model. Ultra-Turrax^®^ also achieved a significantly higher negative predictive value (53.6% [95% CI: 35.1–72.0] vs. 37.5% [22.5–52.5], *p* = 0.0005), while the positive predictive value remained identical (100% [95% CI: 100–100]).

### 3.3. Microbiological Results

Culture positivity varied by specimen type. Ultra-Turrax^®^ produced significantly more positive cultures than the standard method: 70.2% vs. 53.2%; *p* = 0.002, McNemar’s test.

For bone samples, Ultra-Turrax^®^ demonstrated a marked increase in sensitivity (82.6% [95% CI 73.2–92.0] vs. 66.0% [54.4–77.6%] for the standard method, *p* = 0.012). Improvements were smaller and not statistically significant for soft-tissue samples (76.2% vs. 70.2%, *p* = 0.41) and implant fluids (74.3% vs. 68.6%, *p* = 0.36). Specificity exceeded 98% in all subgroups.

Quantitative analysis confirmed the superior performance of Ultra-Turrax^®^: 388 samples showed improved detection vs. 18 favoring the standard method (Table 3). Among 206 samples initially classified as “Rare” or “Few”, Ultra-Turrax^®^ reclassified them as “Numerous” or “Very Numerous”. Overall, Ultra-Turrax^®^ increased bacterial detection in 51% of specimens, compared with 2.4% for the standard technique.

Bacterial counts were significantly higher with Ultra-Turax^®^ (median 4.3 vs. 3.8 log10 CFU/mL, *p* = 0.002), under both aerobic and anaerobic conditions. Inter-method reproducibility was high (κ = 0.84, [95% CI: 0.72–0.95]). All negative controls remained sterile, ruling out cross-contamination during bead-milling or specimen handling.

Among 54 culture-negative samples, 16S rRNA PCR identified bacterial DNA in 14 additional cases (26%) using Ultra-Turrax^®^, compared with 7 cases (13%) using the standard method (*p* = 0.04). Organisms included *S. aureus* (*n* = 3), *S. epidermidis* (*n* = 2), *S. lugdunensis* (*n* = 1), *S. caprae* (*n* = 1), *S. haemolyticus* (*n* = 1), anaerobes (*n* = 3), *E. cloacae* (*n* = 1), *K. aerogenes* (*n* = 1), and *E. coli* (*n* = 1)). Twelve of these fourteen cases showed concordance with clinical and histopathological findings.

### 3.4. Anatomic Site Performance

Ultra-Turrax^®^ yielded the greatest benefit in hip prosthesis infections (*n* = 49), with sensitivity of 83.3% [95% CI 72.1–94.6%] vs. 71.4% [95% CI 57.8–85.1] (*p* = 0.025), confirmed by Bayesian analysis (72.5% vs. 62.1%; *p* = 0.013) (Table 2). Specificity remained 100% [95% CI: 100–100]). Negative predictive value was higher with Ultra-Turrax^®^ (50.0% [95% CI: 23.8–76.2] vs. 36.8% [15.1–58.5], *p* = 0.026).

For knee prostheses (*n* = 25), Ultra-Turrax^®^ showed higher sensitivity (84.2% [95% CI: 67.8–100] vs. 68.4% [47.5–89.3]), but the difference did not reach statistical significance (*p* = 0.083), likely due to limited sample size (Table 2). Bayesian estimates were similar (65.0% [95% CI: 45.8–82.7] vs. 53.1% [34.3–71.9], *p* = 0.054). Negative predictive value was higher for Ultra-Turrax^®^ (66.7% vs. 50.0%), but not statistically significant (*p* = 0.083).

Across all prothesis-associated samples combined, Ultra-Turrax^®^ significantly improved sensitivity (82.5% [95% CI: 73.2–91.9] vs. 68.2% [56.7–79.7], *p* = 0.003), while specificity remained 100% (Table 2). Negative predictive value was also higher (57.7% [38.7–76.7] vs. 42.8% [26.5–59.2], *p* = 0.003), while positive predictive value remained 100%. Notably, Ultra-Turrax^®^ exclusively documented 73 infections, compared with only six detected solely by the standard method (*p* = 0.018; Fisher’s exact test).

### 3.5. Pathogen-Specific Yield

Gram-positive cocci were the most frequently isolated pathogens (*n* = 363, 66.4%), followed by Gram-negative bacilli (*n* = 85, 15.5%), anaerobes (*n* = 80, 14.6%), and Gram-positive bacilli (*n* = 19, 3.5%). No fungi were detected.

Among species, *Staphylococcus aureus* was the predominant microorganism (*n* = 204, 37.3%), followed by *Staphylococcus epidermidis* (*n* = 96, 17.4%), *Pseudomonas aeruginosa* (*n* = 42, 7.7%), and *Cutibacterium acnes* (*n* = 32, 5.9%) (Table 4). Ultra-Turrax^®^ significantly increased the detection of *S. epidermidis* (16% for Ultra-Turrax^®^ vs. 11.3% for the standard method, *p* = 0.026) and *Anaerococcus vaginalis* (1.04% vs. 0%, respectively, *p* = 0.041), though the latter was isolated in only six cases. *S. aureus* was more frequently detected using Ultra-Turrax^®^ (35.3% vs. 30.2%), but the difference did not reach significance (*p* = 0.079). No false positives attributable to cross-contamination were observed.

### 3.6. Time to Positivity

Median time to culture positivity was similar between methods (38 h vs. 40 h, *p* = 0.72). Colony morphology, contamination rate (2.4%), and antibiotic susceptibility profiles were identical, indicating that bead-milling did not impair bacterial viability. In 83% of positive cases, both methods yielded results on the same day. In 17% of dual-positive samples, Ultra-Turrax^®^ provided earlier detection, with time gains ranging from 1 to 14 days. The standard method was faster in only one case (by four days).

Bayesian hierarchical modeling integrating all specimen types confirmed the superiority of Ultra-Turrax^®^, with a posterior probability > 0.995. Model predictions indicated that for every 100 suspected BJIs, Ultra-Turrax^®^ would identify approximately 10 additional true infections without increasing false-positives. Posterior predictive checks demonstrated good model fit (Bayes *p* = 0.67).

## 4. Discussion

This study demonstrates that mechanical bead-milling using the Ultra-Turrax^®^ Tube Drive system significantly enhances microbiological detection in BJIs, particularly from bone specimens. The improvement in sensitivity observed across both culture and molecular methods underscores the critical role of pre-analytical optimization in diagnostic workflows. These findings are especially relevant in low-biomass infections, where conventional vortex homogenization often fails to release bacteria embedded in dense osseous matrices or biofilm structures [20,21,22].

The superiority of Ultra-Turrax^®^ was most evident in bone samples and hip prosthesis infections, where bacterial recovery increased by over 15% compared to the standard method. This aligns with previous studies highlighting the limitations of manual or vortex-based homogenization in bone tissue, which is often poorly penetrated by pathogens and difficult to process due to its mineralized nature [20,21,22,23,24]. Mechanical disruption via bead-milling likely facilitates the release of bacteria from microabscesses, trabecular niches, and biofilm aggregates, improving both culture yield and nucleic acid recovery for molecular diagnostics [23,24,25,26]. The results emphasize the importance of optimizing pre-analytical processing in osteoarticular infection diagnostics.

Our findings are consistent with prior reports on the limitations of classical homogenization and the benefits of sonication and bead-milling techniques in prosthetic joint infections [26,27,28], but extend these observations to native bone infections, an area less explored in the literature. Compared to sonication, Ultra-Turrax^®^ demonstrated superior performance. For instance, a meta-analysis reported global sensitivity and specificity of 79% and 95% after sonication [29], while Hoekstra et al. [30] reported sensitivity and specificity of 80.5% and 97.8%, respectively—figures below those achieved with Ultra-Turrax^®^. Beyond diagnostic performance, our bead-milling process offers practical advantages: reduced aerosol generation, compatibility with standard microbiology workflows, and adaptability to various specimen types without significant tissue loss [31]. The reproducibility of results and absence of cross-contamination further support its safety and robustness.

Importantly, the increased sensitivity did not compromise specificity or contamination rates. The positive predictive value remained at 100%, and the contamination rate was low (2.4%), suggesting that mechanical extraction can be safely integrated into routine diagnostics. Improving the negative predictive value, especially in hip and prosthesis-associated infections, may help reduce diagnostic uncertainty and avoid unnecessary empirical treatments [32].

Enhanced molecular detection with 16S rRNA PCR (26% vs. 13%) suggests that Ultra-Turrax^®^ also improves total DNA recovery, which is crucial for culture-negative infections [23,33]. This dual benefit, higher culture positivity and better molecular sensitivity, positions bead-milling as a valuable upgrade to current protocols, especially in cases with prior antibiotic exposure or chronic biofilm-associated infections.

From a clinical perspective, the ability to detect pathogens more reliably and rapidly has direct implications for patient management. Early and accurate microbiological diagnosis of BJIs remains challenging due to low bacterial burden, previous antibiotic exposure, and biofilm formation [2,13]. In 17% of dual-positives cases, Ultra-Turrax^®^ provided faster results, with time gains of up to 14 days. Early identification of causative organisms enables targeted antimicrobial therapy, reduces reliance on broad-spectrum agents, and may improve outcomes in complex osteoarticular infections.

Nevertheless, some limitations must be acknowledged. The sample size of the knee prosthesis infection subgroup was limited (*n* = 25), restricting the generalizability of conclusions for this subgroup. Over-homogenization may generate heat and shear stress, potentially affecting bacterial viability [34]; however, our protocol optimized parameters to balance efficiency and cell preservation. Additionally, the representativeness of bone biopsies may vary depending on lesion heterogeneity and local ecology. While our dataset is relatively large, further multicenter studies are needed to validate these findings across diverse clinical settings. Future research should also explore the impact of this pre-analytical approach on clinical outcomes. The clinical relevance of polymicrobial detections and low-load isolates warrants further investigation. Although Ultra-Turrax^®^ improved detection of *S. aureus* and coagulase-negative staphylococci, interpreting multiple species in a single sample, particularly distinguishing contamination from true infection, remains a diagnostic challenge [35].

## 5. Conclusions

This study provides strong evidence supporting the integration of Ultra-Turrax^®^ bead-milling into routine diagnostic workflows for osteoarticular infections. Its technical simplicity, high reproducibility, and seamless compatibility with standard microbiology laboratory equipment make it a practical solution for routine use. Future studies should evaluate its impact specifically in prosthetic joint infections, assess its cost-effectiveness in real-world settings, and explore its potential as a standardized pre-analytical step for downstream applications such as metagenomic sequencing. Overall, mechanical bead-milling represents a robust and efficient advance in the microbiological diagnosis of BJIs, with the potential to improve pathogen recovery, refine etiological attribution, and guide more precise and individualized therapeutic strategies.

## Figures and Tables

**Table 1:** Demographic and clinical characteristics of the study population.

	N (%)	Mean, SD ^1^
Age (years)	-	67.61 ± 16.10
BMI ^1^	-	27.08 ± 6.28
Biological data		
Leucocytes (G/L)	-	10.84 ± 5.38
Neutrophils (G/L)	-	8.70 ± 5.92
C-reactive protein (mg/L)	-	119.56 ± 110.21
Type of infection		
Early	49 (42.2)	-
Delayed	32 (27.6)	-
Chronic	32 (27.6)	-
Recurrent	3 (2.6)	-
Prosthetic material		
Hip	49 (42.2)	-
Knee	25 (21.6)	-
Shoulder	2 (1.7)	-
Elbow	2 (1.7)	-
Total prosthetic material	78 (67.2)	-

^1^ SD, standard deviation; BMI, body mass index.

**Table 2:** Diagnostic performance of Ultra-Turrax^®^ versus Standard Method (Global and by sample type). Sensitivity, specificity, and *p*-values from classical and Bayesian analyses for BJI samples. Bayesian latent class modeling was used to address the absence of a perfect gold standard.

			**Conventional**		**Bayesian**		***p*** -Value *
			Estimate	CI 95%	Estimate	CrI 95%	
Global (*n* = 116)	Standard	Sensitivity	75.2%	66.8–83.7	66.7%	57.4–76.3	0.0005
		Specificity	100%	100–100	99.2%	95.7–99.9	
	Ultra-Turrax^®^	Sensitivity	87.1%	80.6–93.7	77.1%	68.4–86.1	
		Specificity	100%	100–100	99.1%	95.3–99.9	
Hip (*n* = 49)	Standard	Sensitivity	71.4%	57.8–85.1	62.1%	48.0–76.0	0.013
		Specificity	100%	100–100	99.1%	95.4–99.9	
	Ultra-Turrax^®^	Sensitivity	83.3%	72.1–94.6	72.5%	58.6–84.7	
		Specificity	100%	100–100	99.2%	95.4–99.9	
Knee (*n* = 25)	Standard	Sensitivity	68.4%	47.5–89.3	53.1%	34.3–71.9	0.054
		Specificity	100%	100–100	99.1%	95.6–99.9	
	Ultra-Turrax^®^	Sensitivity	84.2%	67.8–100	65.0%	45.8–82.7	
		Specificity	100%	100–100	99.2%	95.6–99.9	
On Prosthesis (*n* = 78)	Standard	Sensitivity	68.2%	56.7–79.7	56.1%	44.8–67.3	0.003
		Specificity	100%	100–100	99.1%	95.7–99.9	
	Ultra-Turrax^®^	Sensitivity	82.5%	73.2–91.9	67.7%	56.5–78.3	
		Specificity	100%	100–100	99.1%	95.4–99.9	

* *p*-value, McNemar test comparing performances of Ultra-Turrax^®^ and standard method.

**Table 3:** Semi-quantitative bacterial load categories obtained after Ultra-Turrax^®^ vs. standard homogenization.

		**Ultra-Turrax^®^**				
		**None**	**Rare**	**Few**	**Numerous**	**Very Numerous**
Standard	None	126	65	22	19	2
	Rare	4	75	50	54	18
	Few	1	7	47	68	45
	Numerous	0	3	2	93	45
	Very numerous	0	0	0	1	14

**Table 4:** Pathogen distribution by extraction method. Frequency of bacterial species in bone specimens processed by conventional vortex homogenization vs. Ultra-Turrax^®^.

	**Bacteria**	**Standard Alone (** ***n*** **)**	**Ultra-Turrax^®^ Alone (** ***n*** **)**	**Both (** ***n*** **)**	**Standard (%)**	**Ultra-Turrax^®^ (%)**	***p*** -Value *
Gram-positive cocci	*Streptococcus mitis*/*oralis*	0	1	4	0.70	0.87	1.000
	*Streptococcus pyogenes*	0	0	4	0.70	0.70	1.000
	*Streptococcus agalactiae*	0	0	10	1.74	1.74	1.000
	Other *Streptococcus* spp.	0	2	3	0.52	0.87	0.720
	*Staphylococcus aureus*	1	30	173	30.26	35.30	0.079
	*Staphylococcus epidermidis*	3	30	62	11.30	16	0.026
	Other coagulase-negative *Staphylococci*	1	8	7	1.39	2.61	0.210
	Other *Enterococcus* spp.	4	1	2	1.04	0.52	0.500
	*Enterococcus faecalis*	0	1	15	2.61	2.78	1.000
	*Enterococcus faecium*	0	1	0	0	0.17	1.000
Gram-positive bacilli	*Dermabacter hominis*	0	0	5	0.87	0.87	1.000
	*Corynebacterium* sp.	0	4	5	0.87	1.57	0.420
	*Bacillus cereus*	0	2	3	0.52	0.87	0.720
Anaerobes	*Cutibacterium acnes*	2	7	23	4.35	5.22	0.580
	*Peptostreptococcus* sp.	0	0	10	1.74	1.74	1.000
	*Finegoldia magna*	0	1	9	1.57	1.74	1.000
	*Anaerococcus vaginalis*	0	6	0	0	1.04	0.041
	*Clostridium perfringens*	0	2	0	0	0.35	0.480
	*Bacteroides fragilis*	0	0	5	0.87	0.87	1.000
	Anaerobic polymorphic microflora	1	1	13	2.43	2.43	1.000
Gram-negative bacilli	*Proteus mirabilis*	0	1	15	2.61	2.78	1.000
	*Citrobacter freundii*	0	2	0	0	0.35	0.480
	*Klebsiella pneumoniae*	0	0	25	4.35	4.35	1.000
	*Pseudomonas aeruginosa*	0	9	33	5.74	7.30	0.340

* *p*-value; Chi-square test–comparison between standard protocol and Ultra-Turrax^®^.

## Data Availability

The data supporting the findings of this study are available from the corresponding author upon reasonable request. Statistical analysis was performed by F.S. The file (*20211112_Rapport_Stat_IOAP_Turrax*) has been archived by the Research Directorate of Nîmes University Hospital.

## Abbreviations

The following abbreviations are used in this manuscript:

BJI	Bone and Joints Infection
BMI	Body Mass Index
CA-SFM	Comite de l’Antibiogramme de la Société Française de Microbiologie
95% CI	95% Confident Interval
95% CrI	95% Credible Interval
CRP	C-reactive protein
EBJIS	European Bone and Joint Infection Society
EUCAST	European Committee of Antimicrobial Susceptibility Testing
MCMC	Markov Chain Monte Carlo
SPILF	Société de Pathologie Infectieuse de Langue Française
SD	Standard deviation
